# Predictive value of neutrophil to lymphocyte ratio in patients with acute exacerbation of chronic obstructive pulmonary disease

**DOI:** 10.1371/journal.pone.0204377

**Published:** 2018-09-28

**Authors:** Fei Teng, Huan Ye, Tianjiao Xue

**Affiliations:** Department of Infectious Disease, Fuxing Hospital, Capital Medical University, Beijing, China; Vrije Universiteit Brussel, BELGIUM

## Abstract

**Objective:**

This study aimed to determine the predictive value of the neutrophil to lymphocyte ratio (NLR) in patients with acute exacerbation of chronic obstructive pulmonary disease (AECOPD).

**Methods:**

A retrospective study was conducted from March 2012 to May 2016 in Fuxing Hospital, Capital University of Medical Science. We collected 906 cases (525 males, 381 females, mean age 81.86±9.75 years) diagnosed with AECOPD. The NLR was calculated from their white blood cell (WBC), neutrophil (NEU), and lymphocyte (LYM) counts, which were obtained at laboratory examination.

**Result:**

After treatment, 698 patients with AECOPD improved. The NLR was higher at admission (6.89±6.82) than after treatment (4.19±5.11) (P = 0.000). The area under the receiver operating characteristic curve (AUC) of the NLR for predicting the 28-day mortality rate was 0.737. Using 8.130 as the critical NLR value, the sensitivity was 60.5%, and the specificity was 74.8%. The AUC of the NLR for predicting the frequency of the need for invasive mechanical ventilation was 0.732. Using 10.345 as the critical NLR value, the sensitivity was 54.3%, and the specificity was 84.8%. The AUC of WBC, NEU and LYM for predicting 28-day mortality and the need for invasive mechanical ventilation in these patients were all less than 0.7. An increased NLR was an independent risk factor for 28-day mortality (OR = 1.067, 95% CI = 1.039 to 1.095, P = 0.000), intensive care unit occupancy (OR = 1.046, 95% CI = 1.023 to 1.068, P = 0.000), and the need for invasive mechanical ventilation (OR = 1.042, 95% CI = 1.019 to 1.066, P = 0.000). Compared with those patients without comorbidities, patients with renal dysfunction or upper gastrointestinal bleeding had an increased risk of death within 28 days (OR = 3.102, 95% CI = 1.525 to 6.312; OR = 4.598, 95% CI = 1.825 to 11.583, respectively), ICU admission (OR = 2.228, 95% CI = 1.286 to 3.860; OR = 3.103, 95% CI = 1.402 to 6.866, respectively), and the need for invasive mechanical ventilation (OR = 3.572, 95% CI = 1.822 to 7.000; OR = 4.279, 95% CI = 1.823 to 10.045, respectively).

**Conclusion:**

In patients with AECOPD, the accuracy of the NLR for predicting the 28-day mortality rate and frequency of the need for mechanical ventilation was significantly higher than the accuracy of WBC, NEU and LYM counts. AECOPD patients with an NLR≥8.130 had higher 28-day mortality rate, while those with an NLR ≥10.345 were more likely to need invasive mechanical ventilation.

## Introduction

Chronic obstructive pulmonary disease (COPD) is a common, preventable and treatable disease that is characterized by persistent respiratory symptoms and airflow limitation.[1] COPD is a major cause of morbidity and mortality in the world and is predicted to become the third leading cause of death worldwide by 2020.[2] In 2013, there were 910,809 deaths from COPD in China, accounting for 31.1% of the total deaths from COPD in the world.[3] Acute exacerbation of chronic obstructive pulmonary disease (AECOPD) is a leading cause of hospitalizations in the United States and the major cost driver in COPD.[4] Correctly assessing the severity of AECOPD and providing proper treatment has important clinical significance and socioeconomic benefit.

At present, there is no unified and objective standard for assessing AECOPD severity and its progression using a clinical application. The clinical presentation of COPD exacerbation is heterogeneous; thus, the report from 2017 Global Initiative for Chronic Obstructive Lung Disease (GOLD) and the standards for the diagnosis and treatment of patients with COPD document that were published in 2004 by the American Thoracic Society and the European Respiratory Society both recommend that the severity of the exacerbation should be based on the patient’s clinical signs, and they recommend the following three classifications: Level I: No respiratory failure (treated at home); Level II: Acute respiratory failure-non life threatening (requires hospitalization); and Level III: Acute respiratory failure-life threatening (leads to respiratory failure).[1, 5] Although this classification method can help physicians classify AECOPD patients with various severities in the outpatient department and emergency ward, it is difficult to popularize this system in clinical practice because of the numerous items and difficulty in remembering the criteria. Thus, a rapid and convenient method or biomarker is needed to aid the assessment of severity and individual risk. This area is a primary focus in current research in AECOPD.

The neutrophil to lymphocyte ratio (NLR) is the ratio of neutrophils (NEU) to lymphocytes (LYM) in peripheral blood. The NLR is being increasingly studied as a systemic inflammatory marker, particularly because it is a relatively inexpensive and widely available assessment tool obtained in a routine blood count analysis. The NLR has been studied in the ICU as an inflammatory biomarker. It has a good correlation with the severity of clinical course in patients, in line with APACHE-II and SOFA scores.[6] Studies have identified the NLR as an inflammatory indicator that can effectively predict the prognosis of cancer and cardiovascular diseases.[7–9] In addition, a study of emergency patients showed that the NLR is a better predictor of bacteremia than routine parameters like C-reactive protein (CRP) level, white blood cell (WBC) count and NEU count.[10] An additional two studies on community-acquired pneumonia (CAP) confirmed that the NLR predicts the severity of CAP with a higher prognostic accuracy than is achieved by traditional infection markers, such as WBC, NEU and CRP.[11, 12] In recent years, the NLR has also been investigated as a diagnostic and prognostic marker in COPD. Some studies have evaluated the application of NLR in AECOPD patients, but most of them have used the NLR as an indicator of exacerbation. For example, Mahsuk Taylan et al. found that the NLR is a novel marker of exacerbation in patients with COPD.[13] Until now, no study has investigated whether the NLR can predict the ICU occupancy rate and the frequency of the need for invasive mechanical ventilation in patients with AECOPD. Our retrospective study investigated the predictive value of the NLR in patients with AECOPD.

## Materials and methods

We retrospectively enrolled 906 inpatients with a diagnosis of AECOPD in our hospital from March 2012 to May 2016. The institutional review board approved this study (Fu Xing Hospital, Capital Medical University Institutional Review Board, approval notice number: 2016FXHEC-KY010), and it was conducted in accordance with the Declaration of Helsinki. AECOPD was the reason for hospital admission and was defined as an acute worsening of respiratory symptoms that resulted in additional therapy.[1] Among all patients, 850 were treated with antibiotics after admission, and 56 were non-infected. Patients aged less than 18 years old or with any condition impacting the NEU or LYM count in peripheral blood, such as pregnancy, hematological diseases, or a history of drug use (e.g., granulocyte colony-stimulating factor), were excluded as candidates for this study. In addition, patients with granulocytic deficiency caused by non-inflammatory factors were not registered in our study. All the data included in this study were collected from records of eligible inpatients. The NLR was calculated according to the NEU and LYM counts in routine blood tests. The WBC count, NEU, LYM, monocyte (MON), eosinophil (EOS) and basophil (BAS) counts were all detected on an MEK-8222K automatic hematology analyzer (Nihon Kohden Company). http://dx.doi.org/10.17504/protocols.io.sk6ecze.

### Statistical analyses

Statistical analyses were performed using SPSS version 22. Continuous variables were tested for normality using the Kolmogorov-Smirnov test. Values are presented as the mean±standard deviation. Continuous variables between the data at admission and the data after treatment were compared using a Wilcoxon signed-rank test. Receiver operating characteristic (ROC) curves were constructed for the NLR, WBC, NEU, LYM, MON, EOS and BAS variables to evaluate the 28-day mortality rate, ICU occupancy rate, and the frequency of the need for invasive mechanical ventilation. The areas under the ROC curve (AUC) values with 95% confidence intervals (CI) were calculated and compared with each other. Optimal cut-off values were determined by the Jouden index. Sensitivity and specificity were calculated with 95% CI. Categorical variables were compared using the chi-squared test. The Mann–Whitney U nonparametric test was used to compare continuous variables. Independent risk factors for the 28-day mortality rate, ICU occupancy rate, and the frequency of the need for invasive mechanical ventilation in patients with AECOPD were analyzed using multivariate binary logistic regression. Odds ratios (OR) and 95% CI were estimated. A P value <0.05 was considered significant.

## Results

A total of 906 cases of inpatients diagnosed with AECOPD were collected in this study, including 525 males and 381 females. The mean age of the patients was 81.86±9.75 years old. There were 70 cases of ICU admission and 46 cases of invasive mechanical ventilation; 38 cases died within 28 days.

In this study, 698 patients with AECOPD improved after treatment and were ultimately discharged from the hospital. The NLR variables were assessed using the Kolmogorov-Smirnov test, and the variables did not conform to a normal distribution. The Wilcoxon signed-rank test showed that the NLR was significantly higher when the patients were admitted before any treatment (6.89±6.82) than after they were treated (before they discharged) (4.19±5.11) (P = 0.000).

Within these 698 patients, 34 cases did not receive antibiotic treatment. Accordingly, the patients were divided into infected group (664 cases) and non-infected group (34 cases). The variables NLR and WBC in these two groups were assessed using the Kolmogorov-Smirnov test, and none of the variables conformed to a normal distribution. Therefore, we chose the Wilcoxon signed-rank test from among the nonparametric tests to compare the differences between the data before and after treatment. In the infected group, the NLR was significantly higher before the treatment with antibiotics (7.05±6.94) than after treatment (4.23±5.21) (P = 0.000), and the WBC count was significantly higher before treatment with antibiotics (9.10±4.32) than after they were treated (6.71±2.37) (P = 0.000). In the non-infected group, there was no significant difference in NLR between the patients at the time of admission (3.89±2.02) and at the time of discharge (3.36±2.11) (P = 0.052), and there was no significant difference in WBC between the patients at the time of admission (6.72±3.05) and at the time of discharge (6.16±1.58) (P = 0.304).

To evaluate the 28-day mortality rate, ICU occupancy rate, and the frequency of the need for invasive mechanical ventilation, the ROC curve analysis showed that when NLR, WBC, NEU, MON, BAS, and NEU/WBC ratio were larger, they indicated a more positive test. In contrast, when the LYM, EOS and LYM/WBC ratio were smaller, they indicated a more positive test.

**Fig 1:**
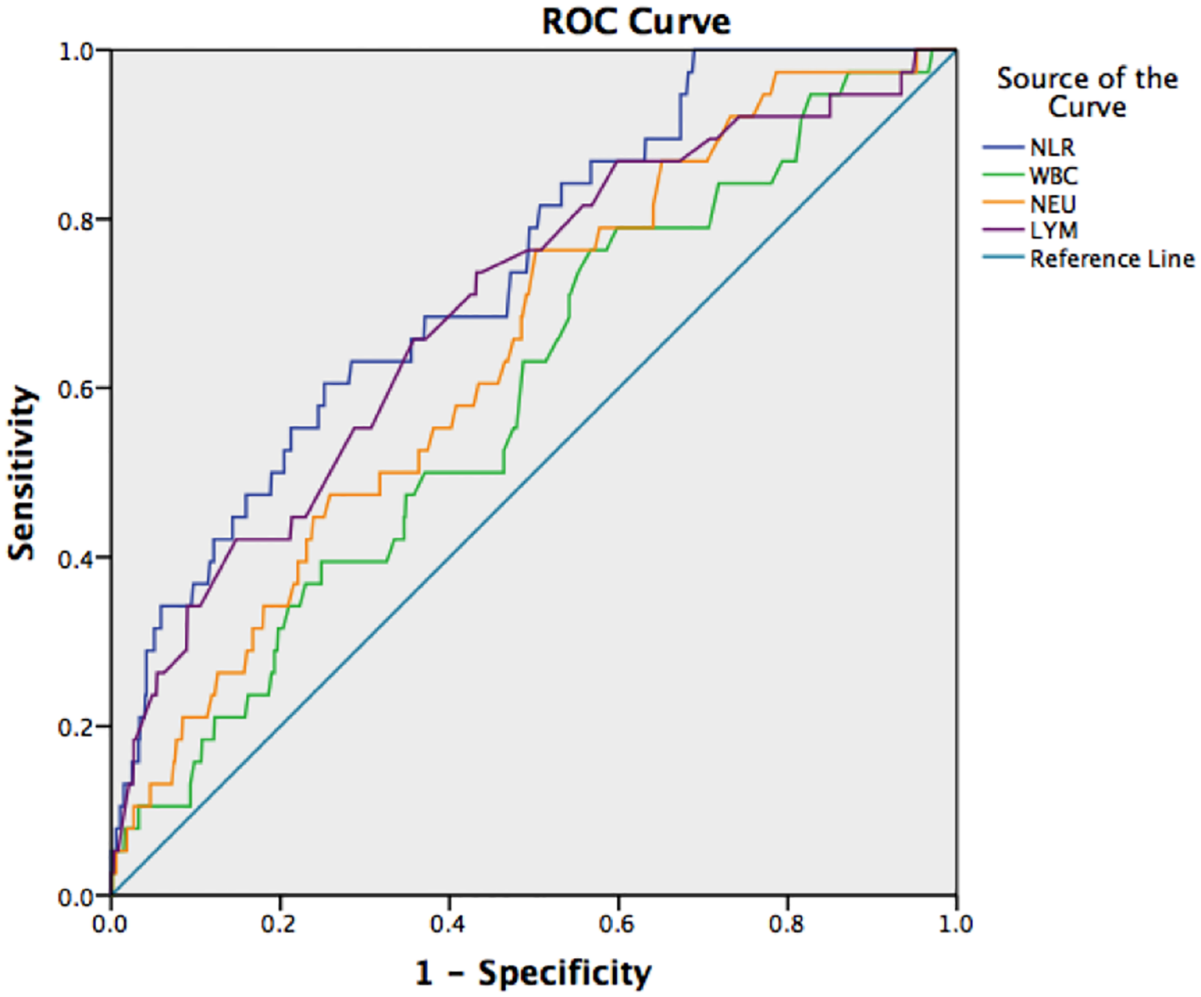
ROC curves of NLR, WBC, NEU, and LYM to predict the 28-day mortality rate.

**Fig 2:**
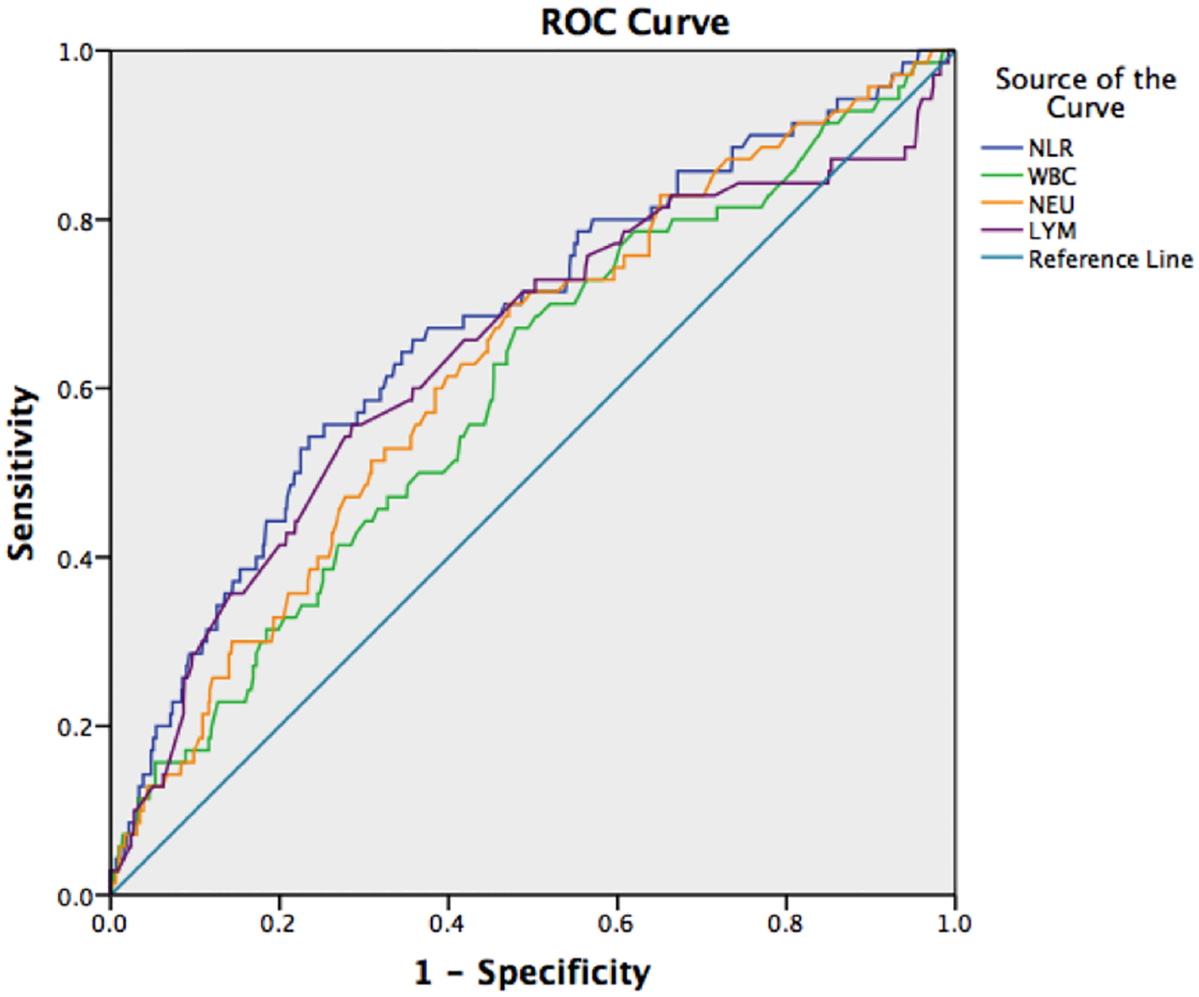
ROC curves of NLR, WBC, NEU, and LYM to predict the ICU occupancy rate.

**Fig 3:**
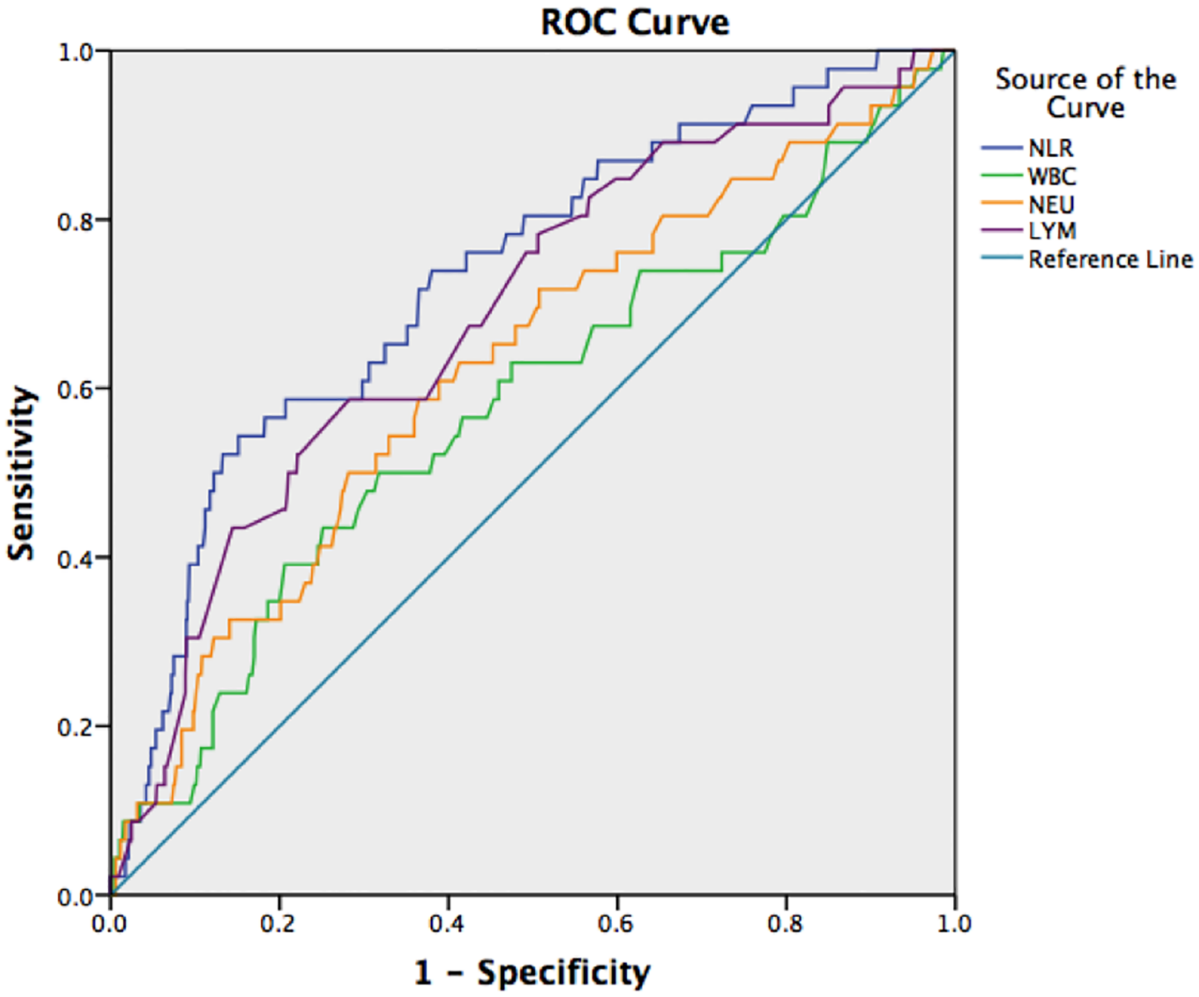
ROC curves of NLR, WBC, NEU, and LYM to predict the frequency of the need for invasive mechanical ventilation.

To predict the 28-day mortality rate in patients with AECOPD, the ROC curve analysis showed that the NLR (when the patients were initially hospitalized) had the highest AUC (0.737) and was followed by the AUC of LYM/WBC (0.736), NEU/WBC (0.729), LYM (0.697), NEU (0.645), BAS (0.623), EOS (0.617), WBC (0.593) and MON (0.512) counts. To predict the ICU occupancy rate in patients with AECOPD, the AUC of NLR was 0.676, the AUC of NEU was 0.629, the AUC of LYM was 0.641 and the AUC of WBC was 0.600. To predict the frequency of the need for invasive mechanical ventilation in patients with AECOPD, the NLR had the highest AUC (0.732) and was followed by the AUC of LYM/WBC (0.729), NEU/WBC (0.701), LYM (0.688), NEU (0.625), WBC (0.581), EOS (0.539), BAS (0.536) and MON (0.520) counts. The results are presented in Figs 1–3 and Tables 1–3.

**Table 1:** AUCs of variables to predict the 28-day mortality rate.

Variable	Area	Sig.	Sensitivity	Specificity	Std.	Asymptotic 95% CI	
			(%)	(%)	Error	Lower	Upper
NLR	0.737	0.000	60.5	74.8	0.039	0.661	0.814
WBC	0.593	0.051	76.3	43.3	0.045	0.505	0.682
NEU	0.645	0.002	76.3	49.8	0.043	0.562	0.729
LYM	0.697	0.000	73.7	56.8	0.045	0.609	0.785
MON	0.512	0.802	13.2	93.8	0.049	0.415	0.609
EOS	0.617	0.014	63.2	64.5	0.049	0.521	0.714
BAS	0.623	0.010	36.8	87.2	0.049	0.526	0.720
NEU/WBC	0.729	0.000	63.2	73.7	0.040	0.650	0.809
LYM/WBC	0.736	0.000	57.9	77.4	0.038	0.661	0.812

**Table 2:** AUCs of variables to predict the ICU occupancy rate.

Variable	Area	Sig.	Sensitivity	Specificity	Std.	Asymptotic 95% CI	
			(%)	(%)	Error	Lower	Upper
NLR	0.676	0.000	54.3	76.6	0.035	0.607	0.744
WBC	0.600	0.005	67.1	52.0	0.036	0.530	0.670
NEU	0.629	0.000	70.0	52.9	0.034	0.562	0.697
LYM	0.641	0.000	55.7	71.4	0.038	0.566	0.716
MON	0.531	0.387	14.3	94.1	0.039	0.455	0.607
EOS	0.553	0.138	50.0	64.6	0.038	0.480	0.627
BAS	0.597	0.007	58.6	61.0	0.035	0.527	0.666
NEU/WBC	0.660	0.000	61.4	68.5	0.036	0.589	0.730
LYM/WBC	0.686	0.000	62.9	68.1	0.035	0.618	0.754

**Table 3:** AUCs of variables to predict the frequency of the need for invasive mechanical ventilation.

Variable	Area	Sig.	Sensitivity	Specificity	Std.	Asymptotic 95% CI	
			(%)	(%)	Error	Lower	Upper
NLR	0.732	0.000	54.3	84.8	0.038	0.656	0.807
WBC	0.581	0.065	39.1	79.4	0.047	0.489	0.672
NEU	0.625	0.004	58.7	63.5	0.044	0.539	0.712
LYM	0.688	0.000	58.7	71.7	0.041	0.609	0.768
MON	0.520	0.651	32.6	78.5	0.049	0.425	0.615
EOS	0.539	0.371	45.7	64.0	0.044	0.452	0.626
BAS	0.536	0.407	21.7	86.6	0.044	0.449	0.623
NEU/WBC	0.701	0.000	69.6	68.1	0.038	0.626	0.776
LYM/WBC	0.729	0.000	54.3	84.9	0.038	0.654	0.804

The NLR was more accurate than WBC, NEU and LYM counts for predicting mortality, ICU admission, and invasive mechanical ventilation. The accuracy of LYM seems to be slightly better than that of NEU. The results of the ROC curve analysis showed that the AUC of NLR for predicting the 28-day mortality rate and the frequency of the need for invasive mechanical ventilation in patients with AECOPD was greater than 0.7. Using 8.130 as the critical NLR to predict 28-day mortality, the sensitivity was 60.5%, and the specificity was 74.8%. Using 10.345 as the critical NLR to predict the frequency of needing invasive mechanical ventilation, the sensitivity was 54.3%, and the specificity was 84.8%.

### Value of predicting the 28-day mortality rate

A total of 906 AECOPD patients included 40 deaths and 38 deaths within 28 days. Accordingly, the patients were divided into a death group (38 cases) and a survival group (866 cases). The sexes of the patients between the death and survival groups were compared using the chi-squared test. The results showed that there was no significant difference in sex between the two groups (P = 0.487).

The variables (NLR, age) of the two groups were assessed using the Kolmogorov-Smirnov test, and none of the variables conformed to a normal distribution. A nonparametric Mann-Whitney U test was performed on the NLR and age between the death and survival groups. The results showed that the differences in the NLR and age between the two groups were significant (P = 0.000, P = 0.004, respectively).

The comorbidities of the patients between death and survival groups were compared using the chi-squared test. Between the two groups, the results identified the following comorbidities: coronary heart disease (P = 0.094), hypertension (P = 0.946), cerebral infarction (P = 0.466), diabetic mellitus (P = 0.694), renal dysfunction (P = 0.000), liver dysfunction (P = 0.008), heart failure (P = 0.001), osteoarthrosis (P = 0.129), atrial fibrillation (P = 0.078), reflux esophagitis (P = 0.298), tumor (P = 0.281), anemia (P = 0.192), gastric (duodenal) ulcer (P = 0.573), upper gastrointestinal bleeding (P = 0.000), hyperlipemia (P = 0.117), and hyperuricemia (P = 0.330). These results are presented in Table 4.

**Table 4:** Baseline characteristics of patients in relation to the 28-day mortality rate.

Variable	Total	Death group	Survival group	Chi-squared test
	(N = 904)	(N = 38)	(N = 866)	P value
Male	525	20	505	0.487
Female	379	18	361	
Coronary heart disease	499	26	473	0.094
Hypertension	623	26	597	0.946
Cerebral infarction	336	12	324	0.466
Diabetic mellitus	288	11	277	0.694
Renal dysfunction	196	18	178	0.000
Liver dysfunction	53	6	47	0.008
Heart failure	181	16	165	0.001
Osteoarthrosis	168	3	165	0.129
Atrial fibrillation	145	10	135	0.078
Reflux esophagitis	243	13	230	0.298
Tumor	115	7	108	0.281
Anemia	186	11	175	0.192
Gastric (duodenal) ulcer	55	1	54	0.573
Upper gastrointestinal bleeding	43	8	35	0.000
Hyperlipemia	296	8	288	0.117
Hyperuricemia	74	1	73	0.330

After screening, the changes in the NLR, age, renal dysfunction, liver dysfunction, heart failure, and upper gastrointestinal bleeding were significant for the 28-day mortality rate in patients with AECOPD. Multivariate binary logistic regression analysis showed that an increased NLR was an independent risk factor for 28-day mortality in patients with AECOPD (OR = 1.067, 95% CI = 1.039 to 1.095, P = 0.000), whereas age (P = 0.052) and liver dysfunction (P = 0.050) were not. Compared with those patients without comorbidities, patients with renal dysfunction (OR = 3.102, 95% CI = 1.525 to 6.312, P = 0.002) or heart failure (OR = 2.537, 95% CI = 1.219 to 5.280, P = 0.013) or upper gastrointestinal bleeding (OR = 4.598, 95% CI = 1.825 to 11.583, P = 0.001) had an increased risk of death within 28 days. These results are presented in Table 5.

**Table 5:** Logistic regression analysis of the 28-day mortality rate.

Variable	B	S.E.	Df	Sig.	Odds Ratio	95% CI for OR	
						Lower	Upper
NLR	0.065	0.013	1.000	0.000	1.067	1.039	1.095
Renal dysfunction	1.132	0.362	1.000	0.002	3.102	1.525	6.312
Heart failure	0.931	0.374	1.000	0.013	2.537	1.219	5.280
Upper gastrointestinal bleeding	1.526	0.471	1.000	0.001	4.598	1.825	11.583

### Value of predicting the ICU occupancy rate

In this study, 906 AECOPD patients included 70 who were admitted to the ICU. Accordingly, the patients were divided into an ICU group (70 cases) and a control group (836 cases). The sexes of the patients between the ICU and control groups were compared using the chi-squared test. The results showed that there was no significant difference in sex between the two groups (P = 0.694).

The variables (NLR, age) in the two groups were assessed using the Kolmogorov-Smirnov test, and no variables conformed to a normal distribution. The nonparametric Mann-Whitney U test was performed on NLR and age to compare the ICU and control groups. The results showed that the difference in the NLR between the two groups was significant (P = 0.000). There was no significant difference in age between the two groups (P = 0.094).

The comorbidities of the patients between the ICU and control groups were compared using the chi-squared test. The results revealed the following comorbidities between the two groups: coronary heart disease (P = 0.399), hypertension (P = 0.466), cerebral infarction (P = 0.992), diabetic mellitus (P = 0.723), renal dysfunction (P = 0.003), liver dysfunction (P = 0.124), heart failure (P = 0.065), osteoarthrosis (P = 0.025), atrial fibrillation (P = 0.053), reflux esophagitis (P = 0.000), tumor (P = 0.989), anemia (P = 0.162), gastric (duodenal) ulcer (P = 0.362), upper gastrointestinal bleeding (P = 0.000), hyperlipemia (P = 0.068), and hyperuricemia (P = 0.744). These results are presented in Table 6.

**Table 6:** Baseline characteristics of patients in relation to the ICU occupancy rate.

Variable	Total	ICU group	Control group	Chi-squared test
	(N = 906)	(N = 70)	(N = 836)	P value
Male	525	39	486	0.694
Female	381	31	350	
Coronary heart disease	500	42	458	0.399
Hypertension	625	51	574	0.466
Cerebral infarction	337	26	311	0.992
Diabetic mellitus	289	21	268	0.723
Renal dysfunction	197	25	172	0.003
Liver dysfunction	53	7	46	0.124
Heart failure	182	20	162	0.065
Osteoarthrosis	168	6	162	0.025
Atrial fibrillation	146	17	129	0.053
Reflux esophagitis	243	33	210	0.000
Tumor	116	9	107	0.989
Anemia	187	19	168	0.162
Gastric (duodenal) ulcer	55	6	49	0.362
Upper gastrointestinal bleeding	44	10	34	0.000
Hyperlipemia	296	16	280	0.068
Hyperuricemia	74	5	69	0.744

After screening, the changes in the NLR, renal dysfunction, osteoarthrosis, reflux esophagitis, and upper gastrointestinal bleeding were significant for the ICU occupancy rate in patients with AECOPD. For the multivariable analysis, we chose Forward: LR as the method. Multivariate binary logistic regression analysis showed that an increased NLR was an independent risk factor for ICU occupancy in patients with AECOPD (OR = 1.046, 95% CI = 1.023 to 1.068, P = 0.000). Compared with those patients without comorbidities, patients with renal dysfunction (OR = 2.228, 95% CI = 1.286 to 3.860, P = 0.004), osteoarthrosis (OR = 0.258, 95% CI = 0.105 to 0.633, P = 0.003), reflux esophagitis (OR = 2.883, 95% CI = 1.704 to 4.879, P = 0.000), or upper gastrointestinal bleeding (OR = 3.103, 95% CI = 1.402 to 6.866, P = 0.005) had an increased risk of ICU admission. These results are presented in Table 7.

**Table 7:** Logistic regression analysis of the ICU occupancy rate.

Variable	B	S.E.	Df	Sig.	Odds Ratio	95% CI for OR	
						Lower	Upper
NLR	0.045	0.011	1.000	0.000	1.046	1.023	1.068
Renal dysfunction	0.801	0.280	1.000	0.004	2.228	1.286	3.860
Osteoarthrosis	-1.355	0.458	1.000	0.003	0.258	0.105	0.633
Reflux esophagitis	1.059	0.268	1.000	0.000	2.883	1.704	4.879
Upper gastrointestinal bleeding	1.132	0.405	1.000	0.005	3.103	1.402	6.866

### Value of predicting the frequency of the need for invasive mechanical ventilation

In this study, 906 AECOPD patients included 46 cases requiring invasive mechanical ventilation (the mode of ventilation is via endotracheal tube). Accordingly, the patients were divided into an invasive ventilation group (46 cases) and a control group (860 cases). The sexes of the patients between the invasive ventilation and control groups were compared using the chi-squared test. The results showed that there was no significant difference in sex between the two groups (P = 0.680).

The variables (NLR, age) in the two groups were assessed using the Kolmogorov-Smirnov test, and no variables conformed to a normal distribution. A nonparametric Mann-Whitney U test was performed on the NLR and age to compare the invasive ventilation and control groups. The results showed that the differences in the NLR and in age between the two groups were significant (P = 0.000, P = 0.035, respectively).

The comorbidities of the patients between invasive ventilation and control group were compared using the chi-squared test. The results showed the following comorbidities between the two groups: coronary heart disease (P = 0.426), hypertension (P = 0.678), cerebral infarction (P = 0.509), diabetic mellitus (P = 0.030), renal dysfunction (P = 0.001), liver dysfunction (P = 0.602), heart failure (P = 0.156), osteoarthrosis (P = 0.325), atrial fibrillation (P = 0.514), reflux esophagitis (P = 0.053), tumor (P = 0.687), anemia (P = 0.001), gastric (duodenal) ulcer (P = 0.162), upper gastrointestinal bleeding (P = 0.000), hyperlipemia (P = 0.023), and hyperuricemia (P = 0.492). These results are presented in Table 8.

**Table 8:** Baseline characteristics of patients in relation to the frequency of the need for invasive mechanical ventilation.

Variable	Total	Invasive ventilation	Control	Chi-squared test
		group	group	
	(N = 906)	(N = 46)	(N = 860)	P value
Male	525	28	497	0.680
Female	381	18	363	
Coronary heart disease	500	28	472	0.426
Hypertension	625	33	592	0.678
Cerebral infarction	337	15	322	0.509
Diabetic mellitus	289	8	281	0.030
Renal dysfunction	197	19	178	0.001
Liver dysfunction	53	4	49	0.602
Heart failure	182	13	169	0.156
Osteoarthrosis	168	6	162	0.325
Atrial fibrillation	146	9	137	0.514
Reflux esophagitis	243	18	225	0.053
Tumor	116	5	111	0.687
Anemia	187	18	169	0.001
Gastric (duodenal) ulcer	55	5	50	0.162
Upper gastrointestinal bleeding	44	9	35	0.000
Hyperlipemia	296	8	288	0.023
Hyperuricemia	74	5	69	0.492

After screening, the changes in the NLR, age, diabetic mellitus, renal dysfunction, anemia, upper gastrointestinal bleeding, and hyperlipemia were significant for the frequency of the need for invasive mechanical ventilation in patients with AECOPD. For the multivariable analysis, we chose Forward: LR as the method. The multivariate binary logistic regression analysis showed that an increased NLR was an independent risk factor for needing invasive mechanical ventilation in patients with AECOPD (OR = 1.042, 95% CI = 1.019 to 1.066, P = 0.000), whereas age (P = 0.340) and anemia (P = 0.114) were not. Compared with those patients without comorbidities, patients with diabetic mellitus (OR = 0.407, 95% CI = 0.180 to 0.924, P = 0.032) or renal dysfunction (OR = 3.572, 95% CI = 1.822 to 7.000, P = 0.000) or hyperlipemia (OR = 0.400, 95% CI = 0.176 to 0.909, P = 0.029) or upper gastrointestinal bleeding (OR = 4.279, 95% CI = 1.823 to 10.045, P = 0.001) had an increased risk of the need for invasive mechanical ventilation. These results are presented in Table 9.

**Table 9:** Logistic regression analysis of the frequency of the need for invasive mechanical ventilation.

Variable	B	S.E.	Df	Sig.	Odds Ratio	95% CI for OR	
						Lower	Upper
NLR	0.041	0.011	1.000	0.000	1.042	1.019	1.066
Diabetic mellitus	-0.898	0.418	1.000	0.032	0.407	0.180	0.924
Renal dysfunction	1.273	0.343	1.000	0.000	3.572	1.822	7.000
Hyperlipemia	-0.917	0.419	1.000	0.029	0.400	0.176	0.909
Upper gastrointestinal bleeding	1.454	0.435	1.000	0.001	4.279	1.823	10.045

## Discussion

Exacerbations of COPD are episodes of worsening of respiratory symptoms that reflect worsening of the underlying chronic inflammation of the airways [14] and result in additional therapy.[15–18] Causes of exacerbations can be divided into bacterial (~50%), viral (~30–60%) and other/unspecified (~15–20%).[19] COPD is associated with chronic inflammation that predominantly affects the lung parenchyma and peripheral airways, resulting in largely irreversible and progressive airflow limitation. This inflammation is characterized by an increase in the number of alveolar macrophages, NEU, T lymphocytes, and innate lymphoid cells.[20] Chronic expectoration, airway obstruction, and a rapid decline in lung function are associated with an increased amount of NEU in the sputum.[21] Allan Klitgaard Sørensen *et al*. found that a low lymphocyte count was a significant predictor of increased mortality in patients with COPD.[22] Therefore, we hypothesized that NEU infiltration and lymphocyte apoptosis may occur in the development of AECOPD. D H Wyllie et al. found that the odds of bacteremia increased with increasing NEU counts and that there was a pronounced increase in bacteremia odds as the lymphocyte count decreased to below 1.5×10^9^/liter.[23] Roser Terradas *et al*.reported that a NLR >7 was an independent marker of mortality in patients with bacteremia.[24]

As an emerging inflammatory indicator, the NLR has been applied in many fields. In recent years, some studies have suggested that the NLR can be used to evaluate acute exacerbation in COPD patients. Seung Jun Lee et al. confirmed that the NLR was significantly higher in patients with COPD exacerbation than in those with stable COPD.[24] Heock Lee *et al*. [25] found that the NLR was inversely associated with the severity of airflow limitation, and the NLR was a significant predictor of exacerbations during a 1-year follow-up period.

In our study, the NLR was significantly higher when the patients were initially hospitalized than after treatment, and this finding indicates that the NLR decreased as the patient’s condition improved. This conclusion is consistent with the research by Mahsuk Taylan *et al*. [13] Their study indicated that traditional markers (CRP, ESR and WBC) were helpful in assessing exacerbated COPD but that the NLR provided more sensitivity. However, when we divided the patients into infected and non-infected groups, we found that in the non-infected group, there was no significant difference in NLR between the patients at the time of admission and at the time of discharge. This finding may indicate that NLR is more valuable in AECOPD induced by bacterial infection.

Noninvasive mechanical ventilation (NIV) is the initial mode of ventilation to treat acute respiratory failure in patients who have been hospitalized for AECOPD because this therapy is thought to be most effective in reducing intubation risk and mortality. In patients who fail NIV as an initial therapy and receive invasive ventilation as subsequent rescue therapy, morbidity, hospital length of stay and mortality are increased.[1] Mihaela S. Stefan et al. also suggested that compared with patients started on NIV and those who were initially intubated, patients who were transitioned from NIV to invasive mechanical ventilation (IMV) had the highest mortality and the longest hospital stays.[26] These results support the use of IMV for critically ill patients. In our study, the result of the multivariate binary logistic regression analysis showed that an increased NLR was an independent risk factor for the frequency of needing IMV in patients with AECOPD. In our study, the AUC of the NLR for predicting the frequency of the need for IMV in patients with AECOPD was 0.732 (>0.7), indicating that its prediction had certain accuracy. The ROC analysis showed that 10.345 was the optimal NLR cut-off, with a sensitivity of 54.3% and specificity of 84.8% for predicting the frequency of the need for IMV. That is, if the NLR is greater than or equal to 10.345, a patient with AECOPD will be more likely to need IMV. Our study confirmed the value of the NLR in predicting the need for IMV, thereby providing a reference for clinicians.

In our study, the result of the multivariate binary logistic regression analysis showed that an increased NLR was an independent risk factor for the 28-day mortality rate in patients with AECOPD. The ROC analysis showed that the AUC of the NLR for predicting the 28-day mortality rate was 0.737 (>0.7), and the accuracy of this evaluation was much better than that of WBC (AUC 0.593) and NEU (AUC 0.645) counts. However, several reports have confirmed the value of WBC and NEU counts for evaluating the prognosis in COPD. Celli *et al*. [27] found that the addition of WBC and NEU significantly improved the ability of clinical variables to predict mortality in patients with COPD. Our study showed that 8.13 was the optimal NLR cut-off, with a sensitivity of 60.5% and specificity of 74.8% for predicting the 28-day mortality rate. That is, if the NLR is greater than or equal to 8.13, a patient with AECOPD is more likely to die. A study by Rahimirad Shaghayegh *et al*. showed that the mortality rate was higher in patients with an NLR ≥4 than in those with an NLR <4. The NLR was independently associated with in-hospital mortality in patients with AECOPD.[28]

In previous studies, indications for respiratory or medical intensive care unit admission included severe dyspnea, changes in mental status, persistent or worsening hypoxemia and/or respiratory acidosis despite supplemental oxygen and noninvasive ventilation, the need for IMV, and hemodynamic instability.[1] In our study, the logistic regression analysis showed that an increased NLR was an independent risk factor for ICU occupancy in patients with AECOPD. Unfortunately, the ROC analysis showed that the AUC of the NLR for predicting ICU occupancy was 0.676 (<0.7), demonstrating that the accuracy of its independent prediction is low. Therefore, the NLR cannot play a role in reducing complexity and cannot be used as an indicator to evaluate whether AECOPD patients need ICU admission.

This study is a retrospective study and has certain limitations. We hope to make further efforts to design a prospective study to explore additional application values of the NLR in patients with AECOPD. The NLR can be obtained directly from a blood routine test and is convenient and relatively inexpensive to perform. The NLR can be quickly acquired without increasing the pain of patients and the burden on clinicians. In an emergency, it can also improve a physician’s accuracy when evaluating the severity and prognosis of AECOPD patients and provides strong support for formulating a treatment plan. The NLR is easy to apply in community hospitals and has bright and extensive application prospects.
